# An Unsupervised Learning Tool for Plaque Tissue Characterization in Histopathological Images

**DOI:** 10.3390/s24165383

**Published:** 2024-08-20

**Authors:** Matteo Fraschini, Massimo Castagnola, Luigi Barberini, Roberto Sanfilippo, Ferdinando Coghe, Luca Didaci, Riccardo Cau, Claudio Frongia, Mario Scartozzi, Luca Saba, Gavino Faa

**Affiliations:** 1 Dipartimento di Ingegneria Elettrica ed Elettronica, Università degli Studi di Cagliari, 09123 Cagliari, Italy; ldidaci@unica.it (L.D.); c.frongia5@studenti.unica.it (C.F.); 2 Laboratorio di Proteomica, Centro Europeo di Ricerca sul Cervello, IRCCS Fondazione Santa Lucia, 00179 Rome, Italy; maxcastagnola@outlook.it; 3 Dipartimento di Scienze Mediche e Sanità Pubblica, Università degli Studi di Cagliari, 09123 Cagliari, Italy; luigi.barberini@unica.it (L.B.); gavino.faa@unica.it (G.F.); 4 Dipartimento di Scienze Chirurgiche, Università degli Studi di Cagliari, 09123 Cagliari, Italy; roberto.sanfilippo@unica.it; 5 UOC Laboratorio Analisi, AOU of Cagliari, 09123 Cagliari, Italy; fcoghe@aoucagliari.it; 6 Department of Radiology, Azienda Ospedaliero Universitaria, University of Cagliari, 40138 Cagliari, Italy; riccardocau00@gmail.com (R.C.); luca.saba@unica.it (L.S.); 7 Medical Oncology Unit, University Hospital and University of Cagliari, 09042 Cagliari, Italy; mario.scartozzi@unica.it; 8 Department of Biology, College of Science and Technology, Temple University, Philadelphia, PA 19122, USA

**Keywords:** unsupervised learning, histopathological images, atherosclerosis, texture analysis

## Abstract

Stroke is the second leading cause of death and a major cause of disability around the world, and the development of atherosclerotic plaques in the carotid arteries is generally considered the leading cause of severe cerebrovascular events. In recent years, new reports have reinforced the role of an accurate histopathological analysis of carotid plaques to perform the stratification of affected patients and proceed to the correct prevention of complications. This work proposes applying an unsupervised learning approach to analyze complex whole-slide images (WSIs) of atherosclerotic carotid plaques to allow a simple and fast examination of their most relevant features. All the code developed for the present analysis is freely available. The proposed method offers qualitative and quantitative tools to assist pathologists in examining the complexity of whole-slide images of carotid atherosclerotic plaques more effectively. Nevertheless, future studies using supervised methods should provide evidence of the correspondence between the clusters estimated using the proposed textural-based approach and the regions manually annotated by expert pathologists.

## 1. Introduction

Stroke is the second leading cause of death and a major cause of disability around the world [1]. According to the World Health Organization, stroke has been responsible for about 6.6 million deaths in 2020. Ischemic stroke is one of the two main subtypes of stroke, accounting for 62.4% of all stroke cases worldwide in 2019 [2]. Between 1990 and 2019, the global number of cases of ischemic stroke increased from 2.04 million to 3.29 million/year and is expected to increase to 4.9 million by 2030 [3]. Projections of the global age-standardized incidence rate of ischemic stroke from 2020 to 2030 indicate an increase in ischemic stroke globally across all age groups [4]. Regarding the pathogenesis of ischemic stroke, the development of atherosclerotic plaques in the carotid arteries is generally considered the leading cause of severe cerebrovascular events. Carotid plaques may be subdivided into two main subtypes: (i) stable plaques and (ii) unstable or vulnerable plaques. Whereas the former are characterized by a predominant fibrous component and abundant collagen deposition, which is at the origin of a thick fibrous cap (>65 microns) and ensures the stability of the plaque [5], vulnerable plaques are characterized by the following phenotype: thin fibrous cap, large lipid-necrotic core, spotty microcalcifications in the fibrous cap, neovascularization inside the plaque, intra-plaque hemorrhage (IPH), hemosiderin granules in the necrotic and fibrous components, monocytes and foamy cells in the fibrous cap [6,7]. These features, taken together, characterize the rupture-prone plaque, whose carriers should be considered “at high-risk subjects” and candidates for surgery [8]. The insurgence of fissurations, erosions and eventually ruptures of the plaque, followed by the exposition of the plaque content to the hemostasis system, forms the basis of local atherothrombosis and thrombo-embolism to the intracerebral vessels, which are the critical events of complicated carotid atherosclerosis, as well the leading cause of symptomatic disease and stroke [9,10,11]. The histological examination of the carotid plaques of patients undergoing endarterectomy has been restricted to the validation of the diagnosis of “plaque instability and vulnerability” performed by radiologists in clinical imaging, including magnetic resonance, computed tomography and ultrasound [12]. In recent years, new reports on the systemic behavior of the atherosclerotic process have reinforced the role of an accurate histopathological analysis of carotid plaques in order to perform the stratification of affected patients and proceed to the correct prevention of complications in other vascular districts [13]. First, carotid atherosclerosis complicated by thrombosis has been associated with an increased risk of subsequent coronary events [14]. This study was confirmed by a report that about 30% of subjects presenting with carotid stenosis due to an occluding atherosclerotic plaque also show a coronary artery stenosis [15]. These recent reports induced the re-evaluation of old papers on the coexistence of carotid and coronary atherosclerosis in 2–14% of patients, a finding that has been forgotten for many years [16]. On this basis, a plaque vulnerability index, mainly based on the histopathology of the removed carotid plaque following endarterectomy, has been proposed as a tool for the prediction of the risk of cardiovascular events in the same subjects [8]. According to this study, subjects with a high vulnerability index emerging from the histopathological study of the carotid plaque should be considered as possible carriers of an unstable, vulnerable plaque in the coronary arteries. These findings, taken together, indicate that the histopathology of the carotid plaques has received much greater attention both in research and in clinical practice, given that a diagnosis of vulnerability at the carotid level may increase the risk of an associated vulnerable plaque in other districts, including coronary arteries.

In this context, the introduction of digital pathology into clinical settings has revolutionized the working environment of pathologists involved in histopathological diagnosis. Furthermore, the broad adoption of whole-slide scanners in digital pathology, together with the automated analysis of tissue characteristics, has evidenced the potential to decrease the workload of pathologists and increase the diagnostic precision, efficiency, and reproducibility of the results. Machine-learning and deep-learning algorithms have simplified the quantitative analysis of very complex images and reduced inter-observer variability [17]. More recently, deep-learning methods have been adopted for several tasks on histopathological images without segmentation and manual annotation. In particular, automated analysis reduces variability and bias by eliminating the need for manual annotation of image features, thereby enhancing the reproducibility. Additionally, automated pipelines facilitate the rapid analysis of whole-slide images, making it easier to evaluate the sample’s morphology and cell composition [18]. Finally, several open-source tools have been developed in the last few years, simplifying the approach to applying machine-learning algorithms to digital pathology [17]. The potential of machine-learning and deep-learning models in digital pathology should encourage pathologists to consider their applications in clinical practice. Overall, computational pathology, especially for those techniques based on whole-slide images and deep learning, is evolving quickly, and several intriguing research challenges must be addressed [19]. In particular, the transition from supervised to unsupervised learning may drastically reduce the time required for manual annotation (which represents an essential yet time-consuming task to produce a large amount of clean data).

The aim of this work is to propose the application of an unsupervised learning approach for the analysis of complex whole-slide images (WSIs) of atherosclerotic carotid plaques to allow a simple and fast examination of their most relevant features. We think such a quantitative analysis might help pathologists enhance their ability to interpret these complex images, making it easier to identify, analyze, and track different regions within the histological images of carotid plaques. To measure and quantify the performance of our tool, which employs clustering to help in the interpretation of whole-slide histological images, we used some metrics that reflect the extent to which the clustering approach is in line with its purpose. In the absence of ground truth annotations by pathologists, which is time-consuming and subject to both high intra- and inter-observer variability, we used two different unsupervised evaluation approaches, namely, the silhouette score and the Davies–Bouldin index. The code developed for the present analysis is available at the following link: https://github.com/matteogithub/Plaques (accessed on 10 June 2024). Finally, as the results from patch-based methods of whole-slide images are dependent on the size of the patches [20], our method was implemented and then used to evaluate the accuracy of the analysis using patches of different sizes. The results were further replicated using two different unsupervised learning approaches.

## 2. Material and Methods

### 2.1. Image Acquisition

Four sections of 5 µm from a single, already scanned and fully anonymized carotid plaque (fixed in 10% buffered formalin, routinely processed, paraffin-embedded, collected on glass slides and stained with hematoxylin and eosin (H&E)) were used to test the proposed approach. The corresponding whole-slide image was obtained using the PrimeHisto XE (Pacific Image Electronics Co., Ltd., New Taipei City 221, Taiwan) scanner and the HistoView software v1.00.50. The WSI scan does not contain any patient data, and no further medical experiments were performed to gather the data.

### 2.2. Image Processing

**Figure 1:**
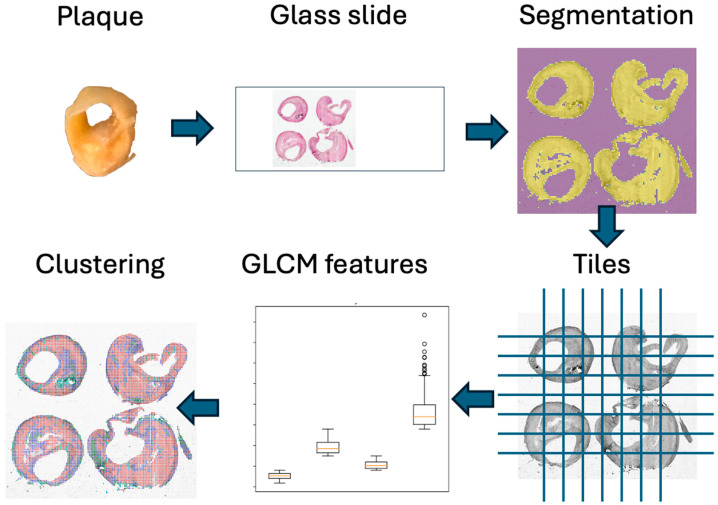
A schematic representation of the steps involved in the analysis.

A schematic representation of the steps involved in the analysis is shown in Figure 1. All the code is freely available and is based on Python with the use of scikit-image [21] (a collection of algorithms for image processing) and scikit-learn [22] (a free and open-source machine-learning library for the Python programming language). The image-processing pipeline was organized into several steps. As a first step, raw images were segmented using Otsu’s thresholding method [23], which calculates an “optimal” threshold by maximizing the variance between two classes of pixels to separate the background from the foreground. In the second step, the foreground was organized into non-overlapping patches of 50 × 50 pixels. In the third step, for each patch separately, the gray-level co-occurrence matrix (GLCM) [24] was computed, which represents a histogram of co-occurring grayscale values at a given offset over an image that allows performing a texture analysis of the underlying image. In the fourth and final step of the processing, from each GLCM matrix, several features were extracted: contrast (a measure of the gray-level contrast), correlation (a measure of gray-tone linear dependencies), energy (used to measure the uniformity of the image) and homogeneity (uniformity of the pixels in the given image).

### 2.3. Clustering

The unsupervised learning task was approached using the k-means clustering algorithm [25,26]. K-means aims to cluster data by separating samples into *n* groups, trying to minimize the within-cluster sum-of-squares. It scales well to large numbers of samples and has been used in a variety of applications in many different fields. However, k-means requires the number of clusters to be specified. We approached this problem using the elbow method [27], which, despite its inherent limitations [28], is a heuristic used in determining the number of clusters in a dataset. In brief, this method consists of the visualization of the explained variation as a function of the number of clusters and choosing the elbow of the curve as the optimal number of clusters to be used. Given the complexity of the original image and the output from the clustering analysis, our approach allows the pathologist to visualize the cluster as an overlay of the grey-scale H&E image, together with the quantitative and graphical analysis of the GLCM feature statistics.

### 2.4. Unsupervised Evaluation

To measure and quantify the performance of our tool, which employs clustering to help in the interpretation of whole-slide histological images, we introduced some metrics that reflect how well the clustering approach accords with its purpose. In the absence of ground truth annotations by pathologists, which is time-consuming and subject to both high intra- and inter-observer variability, we used two different unsupervised evaluation approaches, namely, the silhouette score [29,30] and the Davies–Bouldin index [31]. The silhouette score is specialized for measuring the cluster quality; the best value is 1, and the worst value is −1, whereas values near 0 indicate overlapping clusters. The Davies–Bouldin index is specialized for measuring how well the clustering has been performed using quantities and features inherent to the dataset, and lower index values indicate a better clustering result.

### 2.5. Replication

Due to the known limitations of the k-means algorithm, we performed a replication of the work using Gaussian mixture models (GMMs), which use a probabilistic assignment of data points to clusters. K-means has no mechanism to handle the uncertainty when a data point is close to more than one cluster centroid, fails to produce optimal clusters for complex and non-linear decision boundaries and is sensitive to initial guesses of centroids [32]. Furthermore, k-means can be significantly impacted by a few (or even a single) outliers, which can greatly affect the positions of the centroids and the final clustering [33]. Finally, k-means assumes that the clusters are spherical and have similar sizes, which may not be true for all datasets [34]. A GMM can capture complex data distributions by modeling them as a mixture of Gaussian components.

As the results from patch-based methods (where whole-slide images are organized into patches before applying any analysis) applied to whole-slide images are dependent on the size of the patches [20], our method was implemented and then used to evaluate the accuracy of the analysis using patches of different sizes.

## 3. Results

**Figure 2:**
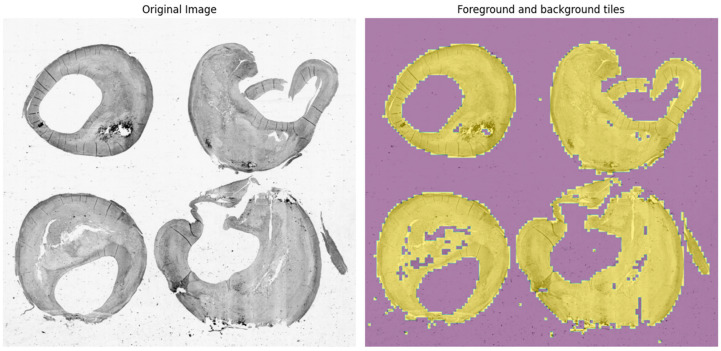
The original grayscale whole-slide image (**left** panel) and the output from Otsu’s thresholding method (**right** panel): in violet, the background, and in yellow, the foreground.

In Figure 2, we show the output from the application of Otsu’s thresholding method on the grayscale original whole-slide image digitized from the glass slice containing four different sections of a carotid plaque from the same subject. The thresholding process has effectively highlighted the boundaries of the foreground within each plaque section. This clear delineation represents an important prerequisite for the subsequent analysis steps, as it allows for the precise identification and extraction of the regions of interest. The segmented foreground is later organized into non-overlapping patches of 50 × 50 pixels. The effect of different sizes used to define the patches on the clustering approach is later reported in this section.

**Figure 3:**
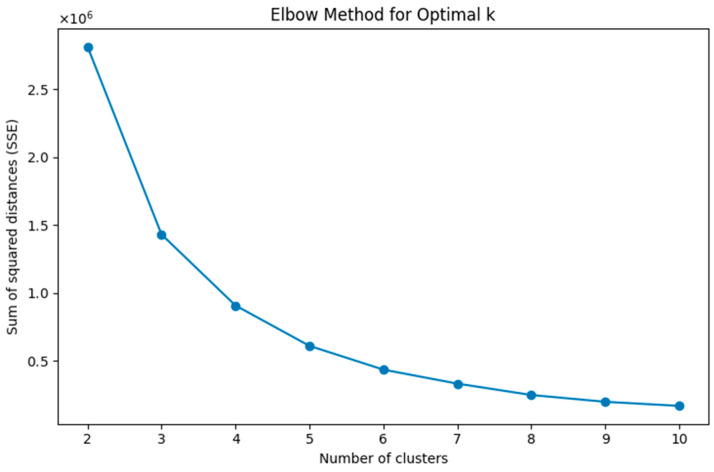
Graphical output from the application of the elbow method.

As k-means requires the number of clusters to be specified, in Figure 3, we show the graphical output from the application of the elbow method, which represents a heuristic used in determining the number of clusters in a dataset. As can be seen, this method consists of the visualization of the explained variation as a function of the number of clusters and suggests choosing the elbow of the curve as the optimal number of clusters to be used. In this case, the optimal k is suggested to be 4.

**Figure 4:**
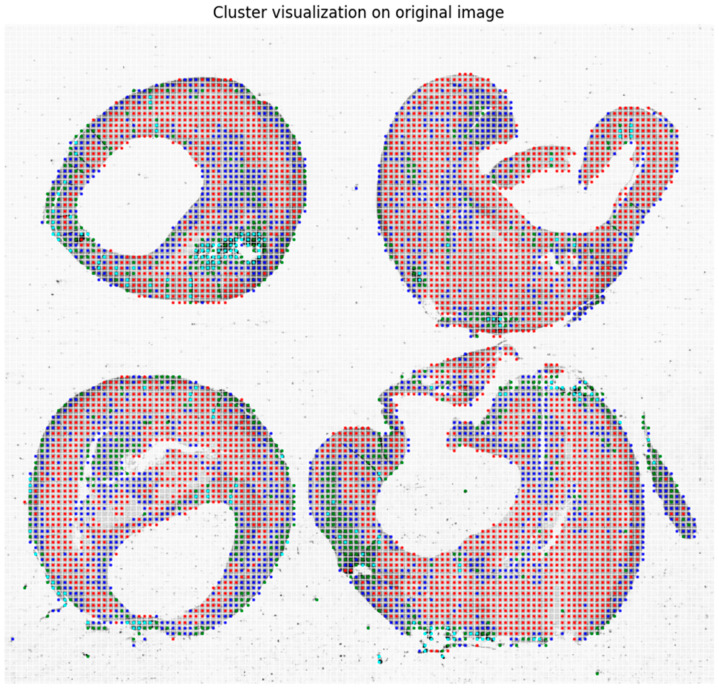
Cluster visualization on the original image, where the segmented foreground is organized into non-overlapping patches of 50 × 50 pixels.

Finally, in Figure 4, the corresponding cluster visualization on the original image allows for highlighting the organization of the different tissues, as described in terms of the GLCM features, of the plaque as defined by the clustering algorithm.

**Figure 5:**
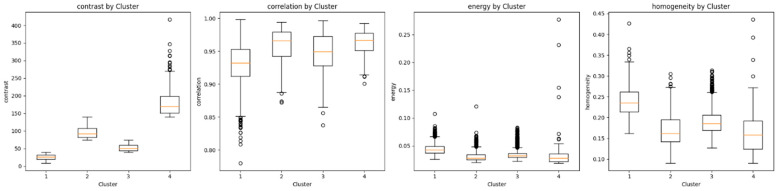
GLCM feature distributions for each identified cluster.

Descriptive statistics and the underlying distributions from the GLCM features, namely, the contrast, correlation, energy and homogeneity, for each cluster separately are reported in Figure 5.

**Figure 6:**
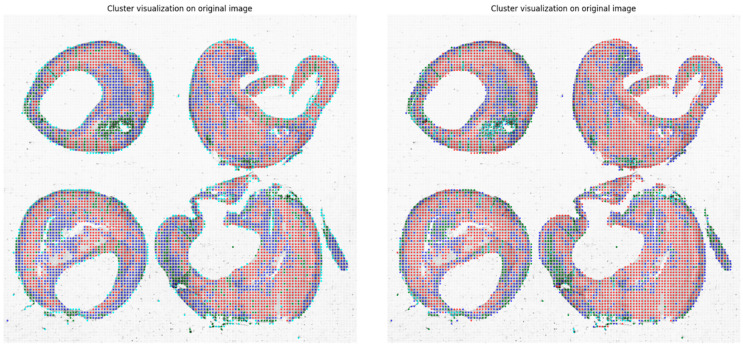
A comparison between the outcomes from the application of the k-means (**left** panel) and GMM (**right** panel) clustering approaches.

**Figure 7:**
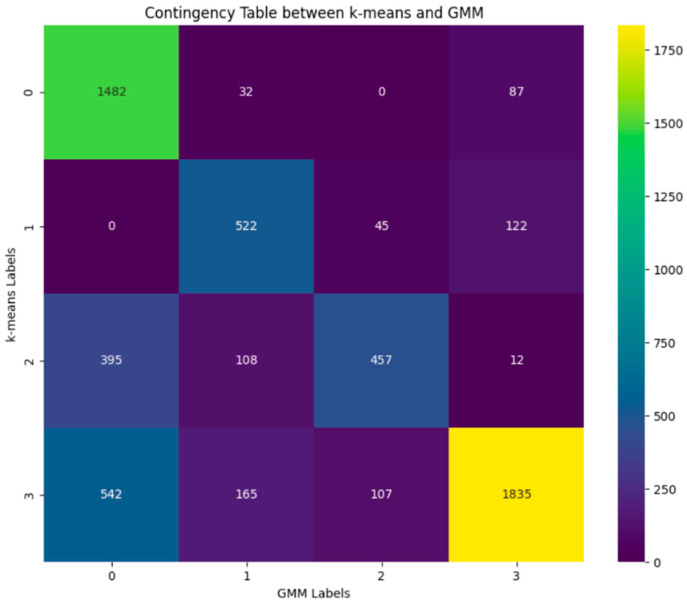
Contingency table that shows the number of data points that are classified similarly between the two clustering results.

As previously described in the Material and Methods section, we used the silhouette score and the Davies–Bouldin index to measure and quantify the performance of the clustering. For the optimal number of clusters k = 4, as evaluated using the elbow method, we obtained a silhouette score of 0.581 and a Davies–Bouldin index of 0.54. A comparison between the two clustering approaches, k-means and Gaussian mixture models, is visually represented in Figure 6. In order to evaluate the similarity between the two clustering procedures, in Figure 7, we show a contingency table that shows the number of data points that are classified similarly or differently between the two clustering results. In this case, we standardized the features by removing the mean and scaling them to the unit variance. The Adjusted Rand Index (ARI) is 0.405 (a value close to 0.0 corresponds to random labelling, independent of the number of clusters and samples, while a value approaching 1 indicates that the clustering outcomes are identical), and the Normalized Mutual Information (NMI) is 0.412 (is between 0 for no mutual information and 1 for perfect correlation).

**Figure 8:**
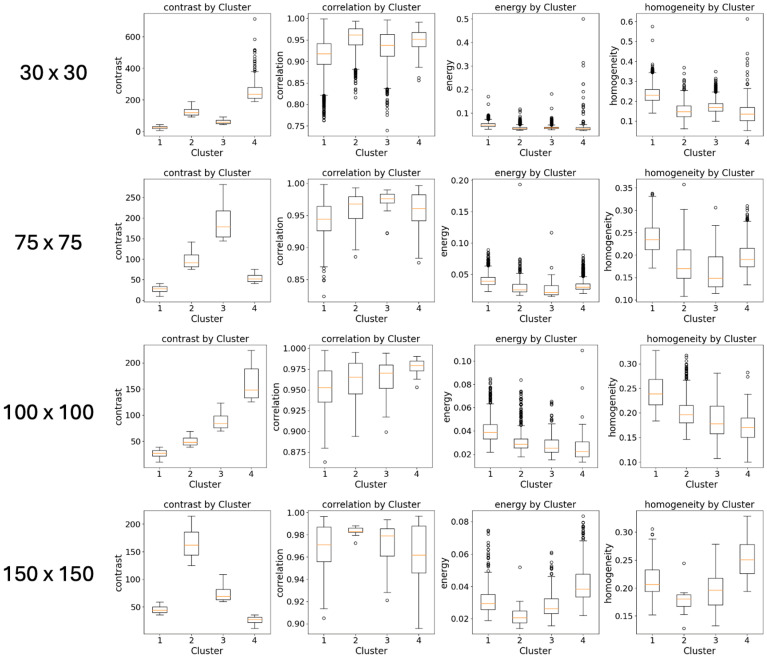
The effect of different sizes used to define the patches on the extracted features.

As described in the Material and Methods section, the results from patch-based methods applied to whole-slide images are dependent on the size of the patches [19]. Therefore, our method was implemented and then used to evaluate the accuracy of the analysis using patches of different sizes. Table 1 and Figure 8 show the effect of different sizes used to define the patches on the clustering approach.

## 4. Discussion

The application of artificial intelligence in carotid plaque detection and characterization has been restricted to non-invasive imaging studies, including computed tomographic angiography (CTA), magnetic resonance imaging (MRI) and ultrasound (US) [35,36,37,38]. In particular, machine learning has been applied to identify vulnerable plaques [6,39]. Lately, deep-learning models based on an artificial neural network have been developed to help clinicians in the diagnosis of plaque vulnerability [40]. All these studies aimed to help radiologists perform an early and accurate prediction of the risk of plaque rupture, allowing appropriate preventive, therapeutic or surgical intervention in order to prevent stroke [41]. Regarding the application of machine learning and deep learning to histological WSI [19,42,43], the vast majority of studies have been focused on the analysis of cancer specimens [44,45]. In this field, of particular interest is a study from the Department of Pathology of the Harvard Medical School in Boston (Boston, MA 02115, USA), in which a new algorithm was evaluated on datasets spanning over 22,000 patient cases, aiming to aid pathologists involved in clinical practice in the diagnosis of rare cancer types [46]. Several other recent studies have shown the impact of adopting artificial intelligence in digital pathology [17,47,48,49,50]. Nevertheless, to the best of our knowledge, only a few studies [41,51] have focused on analyzing histopathological images of atherosclerotic plaques using machine-learning techniques.

Here, we report our findings when applying an unsupervised learning approach to analyze whole-slide images of carotid atherosclerotic plaques. The present approach provides a set of qualitative and quantitative tools to aid pathologists in better investigating the complexity of whole-slide images of carotid atherosclerotic plaques.

Our approach’s qualitative aspect allows pathologists to inspect and interpret the segmented regions within the whole-slide image visually. By applying segmentation techniques, such as Otsu’s thresholding and machine-learning models, we have delineated different tissue types and structures within the plaques. These visual aids are crucial for pathologists as they offer an intuitive understanding of the tissue morphology within the plaques. On the quantitative side, our approach employs feature extraction methods, such as the GLCM, for texture analysis, quantifying the segmented regions’ characteristics. These features, including the contrast, correlation, energy, and homogeneity, provide a detailed numerical description of the tissue properties. Furthermore, the clustering analysis categorizes these features into distinct groups, revealing patterns and relationships within the data that may not be immediately apparent through visual inspection alone.

In particular, the results from the segmentation task allow us to have deep control over the elements of the original image that will be further considered in the following analysis steps. Later, the elbow method will help identify the optimal number of clusters corresponding to the variety of possible types of tissues found within the WSI under investigation. Finally, after the extraction of the underlying textural features and the execution of the clustering method, pathologists may benefit from the graphical visualization where each patch is assigned to the corresponding class, thus providing further help to understand the different types of tissues in the original image. Our tool provides both summary statistics and graphical representations of all the textural features for each cluster and a set of indexes (the silhouette score and the Davies–Bouldin index) that measure the quality of the clustering approach. In the reported experiment, we obtained a number of clusters that are compatible with the main types of tissues that are expected (even if not always present) to be observed in these images: fibrous cap, calcifications, necrotic core, extracellular lipids, intraplaque hemorrhage and inflammations. For the evaluated number of clusters, the computed quality indexes correspond to values (silhouette score of 0.581 and a Davies–Bouldin index of 0.54) that suggest the good quality of the clustering approach.

Moreover, since different clustering algorithms may provide different results because they employ distinct methods and assumptions to group data, it is important to highlight that our results have been further confirmed by a replication analysis, where a different clustering method, namely the Gaussian mixture models, provided evidence of the overall quality of the performed analysis.

Finally, as the results from patch-based methods applied to whole-slide images are dependent on the size of the patches [20], it is relevant to notice that the reported results are consistent over a wide range of sizes used to define the resolution of the analysis, where the number of clusters, the quality of the clustering and the replication results do not change over different values.

## 5. Conclusions

In conclusion, in this study, we show that an approach based on an unsupervised learning method may help pathologists involved in the analysis of very complex whole-slide images of carotid atherosclerotic plaques. In particular, by providing a set of qualitative and quantitative tools, this approach aids pathologists in investigating the complexity of these plaques more effectively, improving the diagnostic procedure and potentially advancing our understanding of atherosclerosis. Future studies using supervised methods should provide evidence of the correspondence between the clusters estimated using the proposed textural-based approach and the regions manually annotated by expert pathologists. However, we think it is important to emphasize that manual annotation is a time-consuming task and that this procedure is subjected to high intra- and inter-observer variability. In our opinion, this latest critical issue still gives more relevance to the reported findings on the use of an unsupervised learning approach.

## Figures and Tables

**Table 1:** The effect of different sizes used to define the patches on the clustering approach. In bold are the results reported in the main text.

Size	K	Silhouette Score	Davies-Bouldin Index	ARI	NMI
30 × 30	4	0.617	0.52	0.362	0.398
**50 × 50**	**4**	**0.581**	**0.54**	**0.405**	**0.412**
75 × 75	4	0.569	0.55	0.273	0.362
100 × 100	4	0.566	0.55	0.438	0.448
150 × 150	4	0.545	0.52	0.264	0.359

## Data Availability

The code developed for the present analysis is available at the following link: https://github.com/matteogithub/Plaques (accessed on 10 June 2024). The original contributions presented in the study are included in the article; further inquiries can be directed to the corresponding author.
